# R-CHOP versus CHOP in HIV-associated lymphoma: a meta-analysis of prospective studies

**DOI:** 10.1186/1750-9378-7-S1-P22

**Published:** 2012-04-19

**Authors:** Ignacio Echenique, Jorge Castillo

**Affiliations:** 1 Department of Medicine, The Warren Alpert Medical School of Brown University, The Miriam Hospital, Providence, RI, USA; 2 Division of Hematology and Oncology, The Warren Alpert Medical School of Brown University, The Miriam Hospital, Providence, RI, USA

## Introduction

Several recent trials have demonstrated superiority with the addition of rituximab to traditional chemotherapeutic regimens in HIV-negative non-Hodgkin lymphoma (NHL) patients. In HIV-positive patients, the benefit of adding rituximab is less clear. In a randomized controlled trial, the addition of rituximab to CHOP showed no survival advantage. We performed a study-level meta-analysis of prospective studies to ascertain outcomes in HIV+ NHL patients treated with CHOP vs. R-CHOP.

## Methods

We performed a Pubmed/MEDLINE literature search from January 1990 through June 2011, with search terms “(HIV OR AIDS) AND lymphoma AND rituximab” and limited our results to English language prospective trials with either CHOP or R-CHOP in HIV+ NHL. Characteristics and outcomes were collected from published data. Chi-square was used to compare the characteristics between groups. The main outcomes were overall response rate (ORR), complete response (CR) rate and 2-year overall survival (OS) and will be reported as odds ratio (OR).

## Results

We identified 3 studies on HIV+ NHL patients treated with R-CHOP and 9 with CHOP from a total of 119 publications. Nine studies (75%) administered *Pneumocytis jirovecci* pneumonia prophylaxis. Four studies (33%) administered prophylactic intrathecal chemotherapy, and in 3 (25%) it was optional. Four studies (33%) administered G-CSF routinely, and 3 studies (25%) only if grade 3/4 neutropenia occurred. A total of 810 patients were studied, 569 treated with CHOP and 241 with R-CHOP. The median age was 38 and 43 years for CHOP and R-CHOP, respectively, with 86% and 85% of male patients, respectively (p=0.98). With regard to HAART, 68% of patients treated with CHOP and 92% with R-CHOP were on HAART prior to lymphoma diagnosis (p<0.0001). The median CD4 count was 109 and 136 cells/mm^3^ in CHOP and R-CHOP patients, respectively. Clinically, 65% and 54% of CHOP patients presented with advanced stage and age-adjusted International Prognostic Index (aaIPI) score 2-3, while the proportion was 74% and 45% in R-CHOP, respectively (p=0.02 for stage and p=0.03 for aaIPI scores). The OR for ORR, CR and 2-year OS in patients treated with R-CHOP vs. CHOP was 1.05 (95% CI 0.71-1.55; p=0.81), 1.42 (95% CI 1.04-1.93; p=0.03) and 2.37 (95% CI 1.73-3.25; p<0.0001), respectively.

## Conclusions

HIV+ NHL patients treated with R-CHOP had higher odds for CR and 2-year OS (42% and 137%, respectively) when compared to CHOP. However, patients treated with R-CHOP also had higher rates of HAART administration, higher CD4 counts and lower aaIPI scores.

